# Management of Complex Root Fractures in Young Patients—Case Series and a Literature Review

**DOI:** 10.3390/jcm13226753

**Published:** 2024-11-10

**Authors:** Hanna Sobczak-Zagalska, Dorota Ogonowska-Paul, Michał Bartmański, Paulina Adamska

**Affiliations:** 1 Department of Pediatric Dentistry, Medical University of Gdańsk, 18 Orzeszkowa Street, 80-204 Gdańsk, Poland; 2 Pediatric Dentistry Clinic of the University Dentistry Center, Medical University of Gdańsk, 18 Orzeszkowa Street, 80-204 Gdańsk, Poland; ogonowska.dorota1@gmail.com; 3 Institute of Manufacturing and Materials Technology, Faculty of Mechanical Engineering and Ship Technology, Gdańsk University of Technology, 11/12 Gabriela Narutowicza Street, 80-233 Gdańsk, Poland; michal.bartmanski@pg.edu.pl; 4 Division of Oral Surgery, Faculty of Medicine, Medical University of Gdańsk, 7 Dębinki Street, 80-210 Gdańsk, Poland

**Keywords:** periodontal ligament, root resorption, tooth discoloration, tooth fractures, tooth injures

## Abstract

**Background:** Root fractures are defined as injuries involving dentine, cementum, and the pulp. They are rare, and they account for 0.5–7% of the dental injuries in the permanent teeth. Root fractures may be the result of direct trauma to the teeth or indirect trauma to the oral cavity. Their incidence is highest in the group of adolescent patients aged 11 to 20 years. The purpose of the paper is to review the literature supported by a case series with three different types of root fractures with various healing patterns, though all with successful long-term treatment outcomes. **Case series:** All presented patients were boys aged 10 to 11 years. Root fractures occurred as a result of direct impact with the teeth by an object. Only one boy had his root-fractured teeth endodontically treated. However, one of the teeth was misdiagnosed with pulp necrosis, and the other became non-vital after additional trauma. **Conclusions:** Root fractures in young patients have good healing potential. Appropriate and early diagnostic and therapeutic procedures for treating root-fractured teeth are necessary to achieve favorable healing and a good long-term prognosis. Regardless of the pattern of healing of fractured roots and the treatment provided, patients require long-term follow-up and the need to plan a management strategy in case complications occur. Failure of the healing of fractured root is a worst-case scenario, especially in patients of developmental age. In such cases, the primary goal of treatment is to preserve the alveolar ridge until growth is complete.

## 1. Introduction

Dental injuries are one of the most common emergencies in the dental office [1] and often require immediate treatment by the dentist. World prevalence of traumatic dental injuries to permanent teeth is 15.2% and to primary ones is 22.7% [2]. Home, parks and kindergarten are the places where dental injuries in young children occur most often [3]. and falls are the dominant cause of their occurrence [4,5,6]. In school-aged children and in adolescent sports activity begins to play a more important role in the etiology of teeth injuries [4,5]. The other common factors resulting in dental injuries are collisions, being hit by an object, traffic accidents and violence [3]. The maxillary central incisors are the teeth that sustain injuries most often. The most common type of trauma in permanent teeth is an uncomplicated crown fracture, while luxations are more common in primary teeth [4,6].

Root fractures are rather rare, comprising 0.5–7% of the injuries affecting the permanent dentition and 2–4% in the primary dentition [7]. The fracture involves the dentin, cementum, and pulp. The injury of the pulp depends on the extent of displacement of the coronal part of the tooth [8]. Root fractures are classified as vertical and horizontal. Depending on the location of the fracture line, transverse fractures are divided into those of the apical third of the root, of the middle third of the root and of the coronal third of the root.

Treatment modalities, like optimal repositioning and flexible splinting of root fractured teeth with displacement of the coronal fragment, have a positive influence upon healing. They favor repairing with hard tissue and pulpal healing. Hence, an appropriate and early diagnosis of the root fracture and the implementation of appropriate therapeutic procedures are very important to minimize the risk of complications [9].

The aim of this paper is to present three different case reports of root fractures in children with good healing efficiency and to offer a review of the current literature about this kind of dental trauma.

## 2. Clinical Cases Presentation

### 2.1. Case Report 1

A generally healthy eleven-year-old boy came to the Pediatric Dentistry Clinic of the Medical University of Gdańsk due to an injury to the upper right and left first incisors (teeth no. 11 and 21 according to the FDI fr. *Federation Dentaire Internationale*). The trauma had occurred the day prior, while riding a bicycle. Immediately after the injury, the patient went to the dental office, where he was provided with initial dental care. The teeth were stabilized with a splint made of a triple twisted wire. The patient was then referred to our clinic.

**Figure 1:**
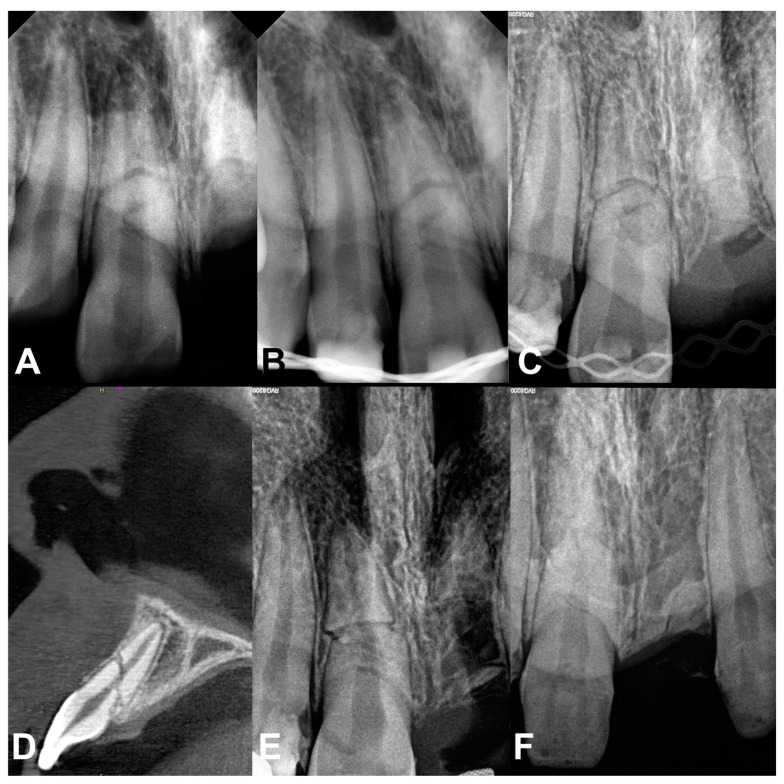
Dental radiographs and CBCT: (**A**) Radiograph after the trauma; (**B**) radiograph 7 days after trauma; (**C**) radiograph 3 months after trauma, teeth splinted with TTS; (**D**)cone beam computed tomography 6 months after trauma; (**E**) radiograph 2 years after trauma; (**F**) radiograph 5 years after trauma.

The patient did not report any spontaneous pain of the injured teeth. An extraoral examination revealed numerous skin abrasions on the right cheek, nose and chin, as well as swelling of the upper lip. The information obtained from the dentist providing initial care showed that upper right central incisor suffered from a root fracture with displacement of the coronal fragment. The upper left central incisor was also root fractured; however, the coronal part was avulsed (Figure 1A).

On the follow-up visit, after 7 days, the boy did not complain of pain of the injured teeth. The percussion test was negative. The pulp reacted positively to the cold stimulus. The radiograph taken showed irregular root fracture lines of tooth 11. There were no signs of inflammation around the left root of the tooth 21 (Figure 1B). A decision was made to replace the rigid wire splint with a flexible one. However, due to the possibility of additional mechanical injuries while removing the splint, its replacement was planned later, after partial stabilization of the broken tooth fragments.

The rigid wires were replaced with a flexible titanium trauma splint (TTS) (Medartis AG, Basel, Switzerland) one month after the injury. TTS was left for another three months. Follow-up visits were continued regularly in accordance with current recommendations (Figure 1C–E). On those visits cold, percussion and radiological examinations did not reveal any pathology.

Five years after the injury tooth 11 was still vital with normal response to percussion and pulp sensibility test. The intentionally retained root 21 was mostly replaced by bone, maintaining the width and height of the alveolar process at this area (Figure 1F).

The patient still requires regular check-ups, which are scheduled every six months. This is due to the unfavorable location of the root fracture in this patient, which can lead to complications more often than in other locations.

### 2.2. Case Report 2

**Figure 2:**
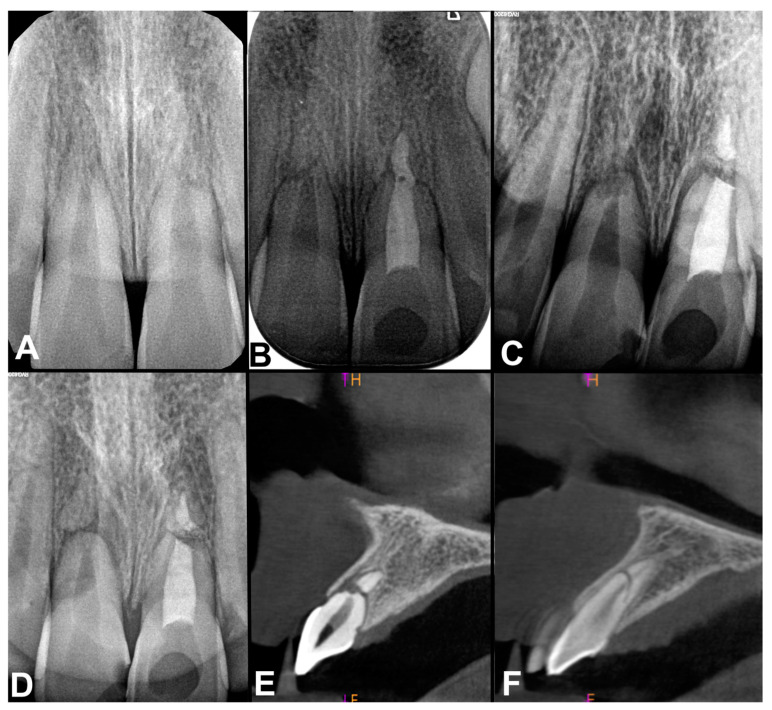
Dental radiographs and CBCT: (**A**) radiograph after the trauma; (**B**) radiograph after root canal treatment of tooth 21 after 4 weeks after trauma; (**C**) radiograph 3 years after the injury—partial luxation (extrusion) of central maxillary incisors; (**D**) radiograph 5 years after trauma—root fracture of tooth 12, inflammatory bone resorption near the fracture line of tooth 11 and the obliteration of apical fragment of the root of tooth 11; (**E**) CBCT 5 years after trauma—inflammatory bone resorption near the fracture line of tooth 11; (**F**) CBCT 5 years after trauma root fracture of tooth 12.

A ten-year-old boy came to the dentist 2 h after suffering trauma of the maxillary central incisors (teeth no. 11 and 21 according to the FDI classification). The injury occurred as a result of falling off a bike. Based on clinical and radiological examination, the dentist diagnosed a fracture in the apical third of the root of the left central maxillary incisor (Figure 2A). The patient did not receive dental first aid as the dentist qualified tooth 21 for endodontic treatment and apical resection. Two days after the accident the boy was referred to the Pediatric Dentistry Clinic of the Medical University of Gdansk. Both maxillary central incisors showed negative responses to pulp sensibility test and tenderness during percussion. A flexible wire (round wire 0.4 mm, Dentaurum, Germany) composite (Flow-Art, Arkona, Poland) splint was applied for 4 weeks.

One week after the trauma, the patient did not complain of pain of the traumatized teeth. Thermal and percussion tests were negative. Two weeks after the accident the boy began to feel pain of the tooth 21 when biting. The percussion test gave a positive response, while a sensibility pulp test was negative.

At the follow-up, after four weeks, tooth 21 was still sensible to percussion test and when biting. Additionally, the crown of tooth 21 turned gray. The trauma splint was removed. The patient was then referred to the endodontist for endodontic therapy of the left central maxillary incisor. The endodontist was asked to treat only the coronal fragment of the tooth. The radiograph taken after root canal filling revealed a root canal filing in both fragments of the tooth and a fracture line in the apical third of the root of the right central maxillary incisor (Figure 2B). Tooth 11 was asymptomatic.

Three years later, the boy had another trauma to maxillary incisors. He fell off his bike. As a result of the fall, the coronal fragments of teeth 11 and 21 were extruded (Figure 2C). Traumatized teeth were repositioned and stabilized for 2 weeks with a flexible wire (round wire 0.4 mm, Dentaurum, Germany) composite (Flow-Art, Arkona, Poland) splint. Lateral incisors and right central one responded positively to cold sensibility pulp test.

Another two years later (five years after the first injury) the crown of tooth 11 turned gray. The radiograph and cone beam computed tomography (CBCT) of this tooth showed inflammatory bone resorption near the fracture line (Figure 2D,E). The root canal in the apical fragment of 11 became obliterated. Right central maxillary incisor was endodontically treated and, although the apical fragment of the root had been filled by the endodontist, there were no signs of inflammation near the fracture line. Radiological examination also revealed a root fracture in tooth 12 (Figure 2D,F), which had not been previously diagnosed or treated and, according to patient, had not been symptomatic.

**Figure 3:**
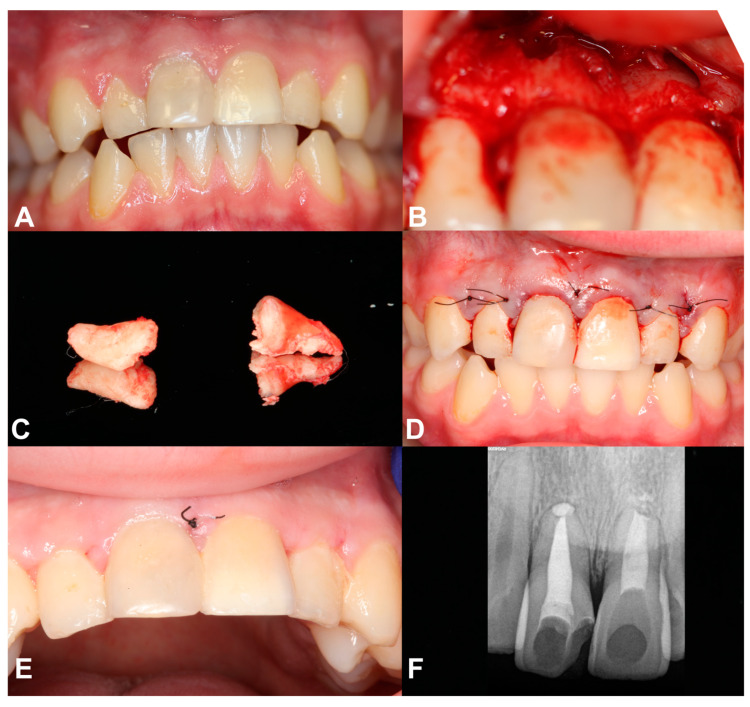
(**A**) Intraoral photography before surgery; (**B**) photography after mucoperiosteal flap was raised and osteotomy was made; (**C**) removed apexes of teeth 11 and 21; (**D**) photography after wound suture; (**E**) healing after 14 days; (**F**) radiography 8 months after surgery (6 years after first injury)—new bone formation is seen.

The patient was referred to the Oral Surgery Clinic of the Medical University of Gdańsk for resection of the apical fragment of teeth 11 and 21. Intraoral photography before surgery was made (Figure 3A). The procedure was performed under a local anesthesia, given via injection. Superior anterior alveolar and nasopalatine nerves were blocked using 4% articaine hydrochloride containing 1:100,000 epinephrine (two ampule; Citocartin 100, Molteni Dental s.r.l., Scandicci (Florence), Italy). Then, envelop incision was made with a no. 15c scalpel blade and a mucoperiosteal flap was raised from tooth 13 to tooth 23 (Figure 3B). Osteotomy was made using a round bur (carbide Lindemann milling cutter HM 408M, Meisinger, Hager and Meisinger GmbH, Neuss, Germany) mounted on a surgical contra-angle handpiece (WS-92 L, W&H Dentalwerk Bürmoos GmbH, Bürmoos, Austria), at 200,000 rpm with abundant irrigation of 0.9% NaCl. Apexes of teeth 11 and 21 were removed automatically using a Heidbrink dental lever (Orimed, Osieck, Poland; Figure 3C). The root canals were prepared retrogradely. Mineral trioxide aggregate (MTA) (Cerkamed, Stalowa Wola, Poland) was putted in the canals. The wound was sutured tightly and without tension (Figure 3D). Non-absorbed nylon sutures 5-0 were used. Hemostasis was achieved. A gauze pad was put on and the patient was instructed to bite on it and hold it for 20 min. The patient was prescribed antibiotics (clindamycin 0.300 g, every 8 h for 5 days). In the peri-operative period, mouthwash with 0.1% chlorhexidine solution twice a day for 14 days was recommended (Eludril Classic, Pierre Fabre Oral Care, Lavaur, France). The sutures were removed on day 14 (Figure 3E).

Eight months after the apicoectomy (six years after the first injury), the radiograph showed bone haling and newly formed bone tissue. The teeth were asymptomatic (Figure 3F).

The patient was informed about the need for further, regular clinical and radiographic follow-up visits once a year.

### 2.3. Case Report 3

A generally healthy ten-year-old boy came to the Pediatric Dentistry Clinic of the Medical University of Gdańsk 3 h after an injury to teeth 11 and 21. The patient was hit with his colleague’s head. There was no loss of consciousness.

**Figure 4:**
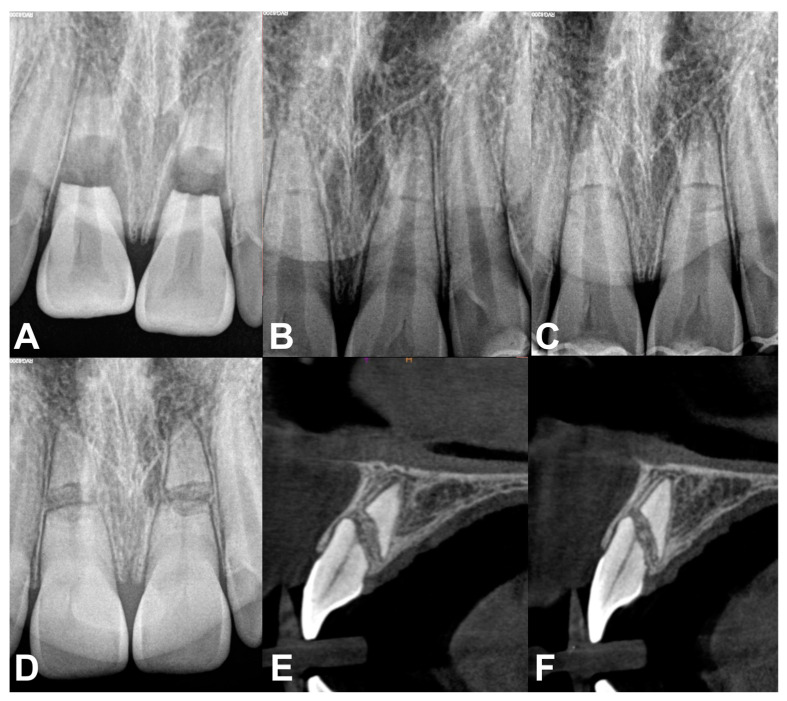
Dental radiographs and CBCT: (**A**) Radiograph taken on the day of the injury; (**B**) radiograph after repositioning and splint application; (**C**) radiograph taken 4 weeks after the injury; (**D**) one year after the injury—bony bridge separating the fragments is seen with a periodontal space around both parts and pulp canal obliteration is present; (**E**) CBCT of the right central maxillary incisor after 14 months post trauma; (**F**) CBCT of the left central maxillary incisor after 14 months post trauma.

The extraoral examination showed no pathology. Intraoral examination revealed palatal displacement of the crowns of the maxillary central incisors. The teeth were tender to percussion and reacted negatively to thermal sensibility test. A preliminary diagnosis of lateral luxation was made. Additionally, the radiographs showed root fractures at the middle third in both traumatized incisors (Figure 4A). Repositioning of the displaced coronal fragments of the central incisors, with only digital pressure, was challenging to perform. Therefore, the teeth were repositioned with forceps and then stabilized with a flexible titanium trauma splint (Medartis AG, Basel, Switzerland) for 4 weeks (Figure 4B).

At follow-up visits after 4 weeks (splint removal) (Figure 4C), 8 weeks and 6 months, the patient did not report any pain. The traumatized teeth were not tender to percussion. The pulp sensibility test for maxillary central incisors was negative. Periotest (Medizintechnik Gulden e.K., Modautal, Germany) results were found to show increased mobility of root-fractured teeth.

After 8 months, the traumatized teeth recovered a positive response to sensibility tests. After one year, periotest measurements demonstrated a decrease in the mobility of the maxillary central incisors. However, clinically, the teeth were still slightly mobile. Normal responses to percussion and pulp sensibility tests were recorded. Radiographically, a bony bridge between the fragments with periodontal space and pulp canal obliteration in both parts of the teeth were seen (Figure 4D–F).

Follow-up visits will be continued yearly for at least 5 years.

## 3. Discussion

This is a case series of three patients with root fractures in permanent teeth. The first patient suffered an oblique root fracture of the right maxillary central incisor with displacement of the coronal part and the fracture line palatally reaching the cervical area. The same patient also suffered a fracture of the root of the left central incisor with avulsion and loss of the coronal fragment. The second patient had multiple injuries over the years. The last patient presented with root fractures with lateral luxation of the coronal parts. In each patient, there were factors that could have a negative impact on the healing of the injured tissues, ranging from unfavorable location of the fracture in the cervical area through inappropriate endodontic treatment of the root-fractured tooth, to a severe displacement of the crown fragments. However, despite these factors, long-term therapeutic success was achieved in each case.

According to the guidelines of the International Association of Dental Traumatology (IADT) the management of root fractures includes the repositioning of the displaced coronal fragment of the tooth and flexible splinting for 4 weeks or 4 months if the fracture line is located cervically [10]. The coronal fragment can be subluxated, extruded, laterally luxated or avulsed. If avulsion occurs, it should be treated in the same protocol as for the avulsed tooth. While the IADT recommends the use of flexible splinting, there is a contrary opinion that the splint should not allow any movement of the coronal fragment during healing period. In this context complete immobilization with rigid splints is important for healing with hard tissue [8,11].

Teeth with root fractures have a good healing potential and a long-term prognosis. However, there are some factors that affect the prognosis of the survival of such teeth. The type of healing turned out to have a strong influence on the prognosis of the root-fractured teeth [12,13]. Therefore, it is suggested that the decision on final treatment should be made after determining the pattern of healing. This usually occurs after 3–6 months of observation. Healing with dentin and cementum (with dental hard tissue) is the most desirable tissues response after root fracture. Fragments are in close contact, and the fracture line is slightly visible on the radiograph. This offers a very good long-term prognosis, regardless of the location of the fracture line [12]. This type of healing occurs in root-fractured teeth with no displacement of the coronal part or with only very minimal displacement. Such a response is also favored by quick and optimal repositioning and by the stabilization of fragments [8].

Interposition of connective tissue occurs mostly in root fractured teeth with displacement of the coronal fragment. According to the studies [13,14,15], this healing pattern is the most common and is associated with the in-growth of connective tissue derived from the periodontal ligament (PDL) into the fracture line. Therefore, there is no connection between the broken fragments. Clinically, this is characterized by increased mobility of the coronal fragment, a slight response to percussion and a normal pulp response to sensibility tests. This type of healing pattern allows for a high tooth survival rate [12,15]. However, a much higher incidence of new injuries was observed in the root-fractured teeth with the interposition of PDL compared with teeth undergoing other types of healing [15]. Radiologically, a line separating the fragments and peripheral rounding of the fracture edges, which are caused by external repair-related resorption, are visible. This type of resorption, also referred to as superficial (external surface resorption (ERS)), is strongly associated with the extrusive luxation of the coronal part and with connective tissue healing. Observation of resorption entities following root fractures allows one to determine some characteristics of these processes. ERS developed approximately 3 months after the trauma and was permanent in nature [16].

In addition to external surface resorption, during the initial phases of intra-alveolar root fracture healing, two other types of resorptions may also occur. These are internal surface resorption (ISR) and internal tunneling resorption (ITR). IRS is seen on the radiograph as a circular radiolucent area centrally in the coronal part of the root canal near the fracture line. As a result of these resorption processes, the fracture edges at the pulpal side of the fracture become rounded. The ITR begins at the fracture line and penetrates behind the predentin layer of the root canal walls in the coronal part, leaving the walls intact [16]. All three resorption entities tend to be self-limiting in 1–2 years after the injury and do not require endodontic treatment [17]. They are strongly related to the healing process by interposition of connective tissue and extrusive luxation of the coronal fragment. However, IRS alone may be a predictor of the union with hard tissue [16].

Interposition of bone and connective tissue is rather rare [13]. This healing pattern occurs in young patients who have sustained an injury before the alveolar bone has fully grown. With the growth of the alveolar process the coronal part of the tooth continues to erupt while the apical part remains in the position it occupied at the time of the injury (Figure 4D–F).

The interposition of inflamed granulation tissue between the fragments is, of course, the worst case scenario. A failure of healing is mostly caused by necrosis and infection of the pulp in the coronal part. Radiological examination usually reveals diastasis of the fracture line, loss of lamina dura and rarefaction of the alveolar bone adjacent to the fracture line. Clinically, the coronal fragment is mobile, slightly extruded and tender to percussion. In this type of tissues response, the location of the fracture was found to have a significant influence on tooth loss. The lowest survival rates were observed for cervical and cervical mid-root fractures [12,18,19]. Modern endodontic materials and techniques allow the opportunity to save teeth with this type of response [15,20].

Monitoring of the pulp condition following root fracture in terms of its survival after trauma should be carried out for at least one year [10]. The initial negative response of the pulp to the sensibility tests alone cannot be an indicator of the necrosis of this tissue. To diagnose pulp necrosis, additional radiographic (i.e., radiolucency within the bone near the fracture line) and clinical symptoms, like tenderness to percussion or extrusion and the loosening and discoloration of the coronal fragment, must occur. These are usually recorded after 2 to 5 months after the injury [21]. In the case of the left maxillary central incisor, in the second presented patient, the decision about the pulp necrosis was made too quickly. Increased sensitivity to percussion was most likely related to connective tissue healing, and coronal discoloration alone was not an indicator of failed revascularization. The study revealed that transient crown discoloration in teeth with root fractures were relatively common and usually indicated a good prognosis of healing. The pulp in the third patient’s root-fractured teeth needed about 8 months to re-innervate and revascularize [22].

It is considered that, in addition to these four short-term responses of tooth tissues to a root fracture, a late response, which is a necrosis and infection of the pulp, should also be mentioned. While in the case of early occurring pulp necrosis, which is caused by contamination of this tissue by bacteria at the time of the injury or shortly after it, the late-occurring necrosis develops as a result of factors not directly related to the root fracture. These factors include caries, broken restoration or periodontal disease, etc. [8].

In cases of early pulp necrosis and in cases where it occurs years after the injury, endodontic treatment of the tooth is recommended. In most cases, such treatment is only required in the coronal part of the tooth, as the apical pulp remains intact and uninfected [23,24]. With early pulp necrosis in root-fractured teeth in patients of developmental age, wide root canals should be expected. Therefore, the pulp lumen in the crown fragment will also be wide, which means that endodontic treatment will require the use of apexification techniques, as in immature teeth [23]. The purpose of such procedures is to create a hard barrier to close a wide-open foramen at the fracture line. It will facilitate obturation of the root canal and prevent the overfilling of material, e.g., gutta-percha. The extrusion of the artificial filling material between the two fragments of the root may lead to persistent inflammation, a foreign body reaction, or delayed healing [8]. Two methods of endodontic treatment of root fractures with wide canals are recommended: (1) a long-term procedure with calcium hydroxide as a canal dressing to induce a calcified barrier and (2) a single-step technique with a hard-setting bio-ceramic material creating an artificial barrier. It is advised to use calcium hydroxide as an intra-canal medication for several weeks before artificial plug application. Both procedures lead to favorable treatment outcomes [25].

In case of root fractures with displacement of the crown fragment, it is recommended to proceed in the same way as in tooth avulsion. However, it is not always possible to replant the crown fragment. In such cases orthodontic or surgical extrusion of the remaining part of the root is an established procedure enabling prosthetic rehabilitation. However, in situations with poor root prognosis, where the crown–root ratio after extrusion is unfavorable and inappropriate for prosthetic reconstruction, extraction is often the treatment of choice. Premature tooth loss in patients of developmental age leads to the growth inhibition of the alveolar bone and to its progressive resorption. As a result, the masticatory system becomes dysfunctional, and prosthetic reconstruction also becomes a problem in the future.

Among the materials and techniques that allow for delaying bone resorption and maintaining or restoring the mass of the toothless alveolar process, leaving the apical fragment of the root seems to be the easiest and most effective procedure [26]. Such an approach, described in the literature as intentional root retention or root submergence, is recommended by IADT as one of the options in crown-root fracture treatment [10,18]. Burying the apical part of the root with vital pulp following the traumatic loss of the coronal fragment of the tooth, is a preferred alternative to the extraction, especially in young patients. Retained root can remain in the bone until an osseointegrated implant can be placed or, provided the appropriate proportions are maintained, it can be used for prosthetic tooth reconstruction. A further reason to consider leaving the root fragment is the possibility of replacement resorption, which gradually leads to the loss of mineralized root tissues and their replacement with bone. Clinical findings from [26] and from our experience with the patient described above indicate that efforts to preserve permanent anterior roots in a young population are justified.

According to IADT guidelines, clinical and radiographic follow-up should be planned after 4 weeks, 6–8 weeks, 6 months, 1 year and then yearly for at least 5 years. In the first year after injury, teeth with root fractures should be reviewed relatively often because this is the time when healing complications are most common and can be detected at an early stage. However, the occurrence of pulp necrosis and infection of the root canal system after root fractures is relatively rare, especially in young patients with wide root canals. This is probably related to better vascularization [8]. Complications of periodontal healing, such as external inflammatory or replacement resorption are not a consequence of a root fracture per se. They are the result of the damage to the PDL and cementum that occurred with partial or complete dislocation of the coronal fragment. [7,8].

## 4. Conclusions

These cases highlight not only how challenging the management of root fractures in developmental patients is, but also the high healing potential of this type of injury and its good long-term prognosis. Following guidelines and undergoing a thorough clinical assessment of the injury are both crucial in making therapeutic decisions and maximizing treatment success.

## Data Availability

The data presented in this study are available upon request from the corresponding author. The data are not publicly available due to privacy restrictions.

## Abbreviations

CBCT	Cone beam computed tomography
ERS	External surface resorption
FDI	Federation Dentaire Internationale
IADT	International Association of Dental Traumatology
ISR	Internal surface resorption
ITR	Internal tunnelling resorption
MTA	Mineral trioxide aggregate
PDL	Periodontal ligament
TTS	Titanium trauma splint
